# Supplementary material for: Topological acoustic triple point
Source: Nat Commun. 2021 Nov 22;12:6781. doi: 10.1038/s41467-021-27158-y (PMC8608830; doi:10.1038/s41467-021-27158-y)
Supplement: Supplementary file 1 — Supplementary Information [file 41467_2021_27158_MOESM1_ESM.pdf]

Supplementary Information for "Topological acoustic triple point"

Sungjoon Park,[1,2,3,*] Yoonseok Hwang,[1,2,3,*] Hong Chul Choi,[1,2] and Bohm-Jung Yang[1,2,3,†]

[1]*Center for Correlated Electron Systems, Institute for Basic Science, Seoul 08826, Korea*
[2]*Department of Physics and Astronomy, Seoul National University, Seoul 08826, Korea*
[3]*Center for Theoretical Physics (CTP), Seoul National University, Seoul 08826, Korea*

CONTENTS

Supplementary Note 1. Proof that isotropic phonon is topological 2

Supplementary Note 2. Euler number as Chern numbers of helicity sectors 4

Supplementary Note 3. Elastic continuum Hamiltonian 5

Supplementary Note 4. k.p Hamiltonian 7

Supplementary Note 5. Linking structure protected by $\mathfrak{q}$ 9

Supplementary Note 6. Nodal lines 11

Supplementary Note 7. Details of the 3D Lieb lattice model 12

Supplementary Note 8. The effect of boundary condition on surface localized states 14

Supplementary Note 9. Phonon angular momentum at the surface 17

This Supplementary Information is organized as follows. Supplementary Note 1 provides a proof that isotropic phonon is topological. We show that the relevant topological charge $\pi_2(O(3)/O(1)\times O(2))$ is given by the tuple $\mathfrak{q}{=}(\mathfrak{n}_{\rm sk},\mathfrak{e})$, where $\mathfrak{n}_{\rm sk}$ is the skyrmion number and $\mathfrak{e}$ is the Euler number. We also show that $\mathfrak{e}{=}2\mathfrak{n}_{\rm sk}$. In Supplementary Note 2, the Euler number of isotropic phonons ($\mathfrak{e}{=}2$) is explained in terms of the Chern numbers of eigenstates in the helicity sectors. In Supplementary Notes 3 and 4, we discuss effective Hamiltonians describing the the topological acoustic triple points (TATP)s. In Supplementary Note 3, we focus on the elastic continuum theory which describes the acoustic phonons, and in Supplementary Note 4, we discuss the k.p theory at high-symmetry points. In Supplementary Note 5, we prove that under a $\mathcal{PT}$ symmetric perturbation that breaks the triple degeneracy of a TATP, the TATP evolves into nodal lines with a linking structure. We also provide the parameters used to obtain the linking structures shown in Figs. 4 and 5 in the main text. Supplementary Note 6 discusses nodal lines emanating from the TATP. Especially, our convention for counting nodal lines, which is used to relate the Euler number and the number of nodal lines, is precisely defined. In Supplementary Note 7, we provide details on the 3D Lieb lattice model. In Supplementary Note 8, we study how boundary conditions affects the appearance of surface localized states. We compare the surface states with the Dirichlet and free boundary conditions. In Supplementary Note 9, we discuss the phonon angular momentum accumulated at the edges from the phonon angular momentum Hall effect, by separately considering the contributions from the bulk and the surface localized modes.

* These authors contributed equally to this work.

† Electronic address: bjyang@snu.ac.kr

Supplementary Note 1. PROOF THAT ISOTROPIC PHONON IS TOPOLOGICAL

In this Supplementary Note, we study the topological charge q in detail. We also refer the readers to Refs. 1–3, where similar results were also obtained. As in the main text, we use the dynamical matrix of isotropic phonon as the model Hamiltonian, whose matrix components are given by $[D_{\mathbf{k}}]_{\alpha\beta} = v_T^2 k^2 \delta_{\alpha\beta} + (v_L^2 - v_T^2) k_\alpha k_\beta$.

In general, when the system has both the time reversal symmetry $\mathcal{T}$ ($\mathcal{T}^2 = 1$) and the inversion symmetry $\mathcal{P}$ ($\mathcal{P}^2 = 1$), or the combined symmetry $\mathcal{PT}$, the Hamiltonian can always be chosen to be real by choosing the gauge in which $\mathcal{PT} = \mathcal{K}$, where $\mathcal{K}$ is the complex conjugation operator. Because there is also a gap between the longitudinal and the transverse modes away from $\mathbf{k} = 0$, the Hamiltonian can be written as $\epsilon^T_{\mathbf{k},L}\epsilon_{\mathbf{k},L} - \epsilon^T_{\mathbf{k},T_1}\epsilon_{\mathbf{k},T_1} - \epsilon^T_{\mathbf{k},T_2}\epsilon_{\mathbf{k},T_2}$ after sending the energy of the longitudinal mode (L) to 1 and the transverse modes (T_1 and T_2) to -1. Equivalently,

$$H_{\mathbf{k}} = E^T_{\mathbf{k}} \begin{pmatrix} 1 & 0 & 0 \\ 0 & -1 & 0 \\ 0 & 0 & -1 \end{pmatrix} E_{\mathbf{k}}, \quad E_{\mathbf{k}} = \begin{pmatrix} \epsilon_{\mathbf{k},L} \\ \epsilon_{\mathbf{k},T_1} \\ \epsilon_{\mathbf{k},T_2} \end{pmatrix}. \tag{1}$$

Since $E_{\mathbf{k}} \in O(3)$ and the Hamiltonian is invariant under $E_{\mathbf{k}} \to F_{\mathbf{k}} E_{\mathbf{k}}$ with $F_{\mathbf{k}} \in O(1) \times O(2)$, the topological charge of the triple point at $\mathbf{k} = 0$ can be characterized by the second homotopy group of the classifying space $O(3)/[O(1) \times O(2)]$.

Because we choose a base point when computing the homotopy, we can assume that the classifying space B is connected to the identity: $B = SO(3)/S[O(1) \times O(2)]$, where $S[O(1) \times O(2)]$ is the subgroup of $O(1) \times O(2)$ with unit determinant. The space $SO(3)/S[O(1) \times O(2)]$ can be viewed as a fiber bundle with base space $B = SO(3)/S[O(1) \times O(2)]$, total space $E = SO(3)$, and fiber $F = S[O(1) \times O(2)]$. It is useful to note that F consists of two types of matrices, once we choose a representation of F:

$$\begin{pmatrix} 1 & 0 & 0 \\ 0 & \cos\theta & \sin\theta \\ 0 & -\sin\theta & \cos\theta \end{pmatrix}, \quad \begin{pmatrix} -1 & 0 & 0 \\ 0 & -\cos\theta & -\sin\theta \\ 0 & -\sin\theta & \cos\theta \end{pmatrix}. \tag{2}$$

Therefore, F has two connected components F^+ and F^-, characterized by the sign of the determinant of $O(1)$, or equivalently the sign of the determinant of $O(2)$, which is ± 1, which corresponds to $F^\pm$. We are interested in computing $\pi_n(B, b_0)$, where $b_0 \in B$ is the base point. To do this, we can examine the exact sequence for fibration [4]:

$$\cdots \to \pi_n(F, x_0) \to \pi_n(E, x_0) \xrightarrow{p_*} \pi_n(B, b_0) \to \pi_{n-1}(F, x_0) \to \cdots \to \pi_0(E, x_0), \tag{3}$$

where p is a projection map $p : E \to B$ and $x_0 \in F = p^{-1}(b_0)$. Because we fix the base point when computing the homotopy group, we can take F to be the component connected to the identity, i.e. F^+, which is topologically equivalent to the group $SO(2)$ (or equivalently, a circle). We find it useful to note that the above sequence is actually a consequence of the following exact sequence for pairs because the projection map p induces the isomorphism $p_* : \pi_n(E, F, x_0) \to \pi_n(B, b_0)$ for $n \geq 1$:

$$\cdots \to \pi_n(F, x_0) \xrightarrow{i_*} \pi_n(E, x_0) \xrightarrow{j_*} \pi_n(E, F, x_0) \xrightarrow{\partial} \pi_{n-1}(F, x_0) \to \cdots \to \pi_0(E, x_0) \tag{4}$$

Here, the map i_* and j_* are induced by the inclusions $i : (F, x_0) \hookrightarrow (E, x_0)$ and $j : (X, x_0, x_0) \hookrightarrow (X, F, x_0)$. The map ∂ is induced by restricting the map $(D^n, S^{n-1}, s_0) \to (E, A, x_0)$ to S^{n-1}, where D^n is the n-dimensional disk, S^{n-1} is the $(n-1)$-dimensional sphere, and s_0 is a point in S^{n-1}.

The part of the above sequence that we need is:

$$\cdots \pi_2(E, x_0) \xrightarrow{j_*} \pi_2(E, F, x_0) \xrightarrow{\partial} \pi_1(F, x_0) \xrightarrow{i_*} \pi_1(E, x_0) \cdots \tag{5}$$

Using the homotopy data [5] for $SO(n)$, this sequence becomes

$$\cdots 0 \xrightarrow{j_*} \pi_2(E, F, x_0) \xrightarrow{\partial} \mathbb{Z} \xrightarrow{i_*} \mathbb{Z}_2 \cdots \tag{6}$$

Thus, $\pi_2(E, F, x_0) = 2\mathbb{Z}$, and ∂ is an injective map of $\pi_2(E, F, x_0)$ into the kernel of i_*. Thus, nontrivial elements of $\pi_2(E, F, x_0)$ can be characterized by maps $r : (D^2, S^1, s_0) \to (E, F, x_0)$ such that S^1 is mapped to $F = SO(2) \cong S^1$ with two windings.

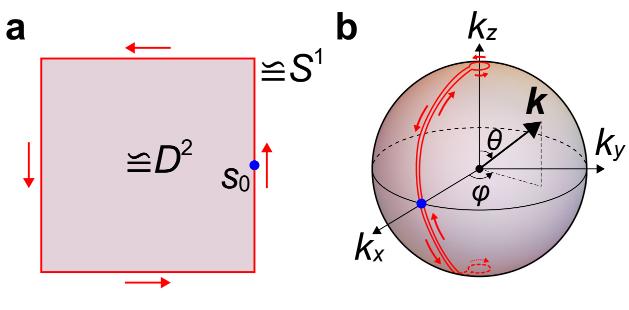

Supplementary Figure 1. **Evaluating the topological charge.** **a,** Illustration of D^2, its boundary S^1 (red line), and the base point s_0 (blue dot). The orientation of the boundary is indicated with the red arrows. **b,** D^2 can be mapped to the sphere as shown. The points to which S^1 is mapped is indicated by the red line with the orientation indicated by the red arrows.

Using this, we can prove that the acoustic phonon modes are topologically nontrivial. The unit normalized phonon po-

larization vectors are

$$\epsilon_{\mathbf{k},L} = \frac{1}{k}(k_x, k_y, k_z) \tag{7}$$

$$\epsilon_{\mathbf{k},T_1} = \frac{1}{\tilde{k}}(-k_y, k_x, 0) \tag{8}$$

$$\epsilon_{\mathbf{k},T_2} = \frac{1}{\tilde{k}k}(-k_x k_z, -k_y k_z, k_x^2 + k_y^2). \tag{9}$$

The goal is to compute the topological charge $\pi_2(B, b_0) \cong \pi_2(E, F, x_0)$ defined on a sphere S^2 with origin at $\mathbf{k} = 0$ in the momentum space. First, we note that

$$E_{\mathbf{k}} = \begin{pmatrix} \epsilon_{\mathbf{k},L} \\ \epsilon_{\mathbf{k},T_1} \\ \epsilon_{\mathbf{k},T_2} \end{pmatrix} \in SO(3). \tag{10}$$

Since D^2 can be identified with the sphere in the momentum space as illustrated in Supplementary Figure 1a, $E_{\mathbf{k}}$ can be viewed as a map from D^2 to $SO(3)/S[O(1) \times O(2)]$ which is well-defined everywhere on D^2. (When we view $E_{\mathbf{k}}$ as a map from the sphere to E, singularities arise at the N and S poles.)

Now, let us consider the following rotation matrix

$$R_{\mathbf{k}}(t) = \begin{pmatrix} \cos t(\theta - \frac{\pi}{2}) & 0 & \sin t(\theta - \frac{\pi}{2}) \\ 0 & 1 & 0 \\ -\sin t(\theta - \frac{\pi}{2}) & 0 & \cos t(\theta - \frac{\pi}{2}) \end{pmatrix} \tag{11}$$

where $\mathbf{k} = k(\sin\theta\cos\phi, \sin\theta\sin\phi, \cos\theta)$. Notice that $R_{\mathbf{k}}(t)$ is nothing but the rotation about the y axis by the angle $t(\theta - \frac{\pi}{2})$. When $t = 0$, we see that it is the identity map, while for $t \neq 0$, it is a continuous function of θ on S^2. Therefore, $E_{\mathbf{k}}(t) = E_{\mathbf{k}} R_{\mathbf{k}}(t)$ defines a continuous deformation of an element in $\pi_2(B, b_0)$. Here, we note that the energy gap between the longitudinal and the transverse modes is always preserved under this transformation. Also, because the deformation is identity at $\theta = \pi/2$, the base point is preserved under this deformation if we choose x_0 to be the identity matrix in $SO(3)$, to which s_0 is mapped under $E_{\mathbf{k}}$, see Supplementary Figure 1. Now, $E_{\mathbf{k}}(1) = E_{\mathbf{k}} R_{\mathbf{k}}(1)$ is an element of the component connected to the identity in F as we trace along S^1 (the boundary of D^2), so that $E_{\mathbf{k}}(1) \in \pi_2(E, F, x_0)$. Therefore, the topological charge $\pi_2(B, x_0) \cong \pi_2(E, F, x_0)$ can be computed by counting the winding number in F of the map $E_{\mathbf{k}}(1)$ when $\mathbf{k}$ is restricted to S^1 (the boundary of D^2): Near the N pole, we have

$$E_{\mathbf{k}}(1) = \begin{pmatrix} 1 & 0 & 0 \\ 0 & \cos\phi & \sin\phi \\ 0 & -\sin\phi & \cos\phi \end{pmatrix}, \tag{12}$$

and near the S pole, we have

$$E_{\mathbf{k}}(1) = \begin{pmatrix} 1 & 0 & 0 \\ 0 & \cos\phi & -\sin\phi \\ 0 & \sin\phi & \cos\phi \end{pmatrix} \tag{13}$$

while $E_{\mathbf{k}}(1)$ is constant along the line connecting the north and the south poles. Since the north pole is traversed counterclockwise while the south pole is traversed clockwise, we see that S^1 winds twice in $F \cong S^1$, i.e. the charge is $2 \in \pi_1(F, x_0)$. As we explain below, this topological charge is nothing but the Euler number [6, 7] $\mathfrak{e}$ of the transverse modes. Let us also observe that the longitudinal mode has nonzero skyrmion number $\mathfrak{n}_{\mathrm{sk}}$.

To make the connection to the Euler number $\mathfrak{e}$, let us begin by noting that the space $B = SO(3)/S[O(1) \times O(2)]$ can be thought of unoriented planes embedded in $\mathbb{R}^3$. Because all vector bundles over S^2 can be oriented [8], we can choose a map from S^2 to B to lie in $B^+ = SO(3)/F^+ = SO(3)/[SO(1) \times SO(2)]$, which is nothing but the space of oriented planes. Alternatively, we can also choose the map to lie in $B^- = SO(3)/F^-$, which will be discuss later. Since oriented planes are determined by an ordered pair of orthonormal vectors, the fiber bundle can be identified with the sphere bundle (fiber bundle with fiber S^1). It is well known that such bundles are characterized by the Euler number. The Euler number can be computed by choosing a section, which can always be done over $S^2 - \{x_1, ..., x_k\}$ for finite non-negative integer k (sphere minus finite number of points x_i), and counting the winding number around the points at which the section is not well-defined [9]. This is essentially what we have done during the computation of $\pi_2(B, x_0)$. Another simple way to see that the transverse modes must be characterized by the Euler number is that the transverse modes form a basis for the tangent space to the sphere S^2.

Because we choose an ordered basis for the planes, normal vector to the oriented plane is fixed by the oriented plane through the cross product of the ordered basis (and vice versa). The normal vector to the oriented plane is nothing but the line bundle, and because the line bundle on S^2 is always trivial [8], we can choose a global section. Because such a section is a map from S^2 (sphere in the momentum space) to S^2 (normalized longitudinal mode), its topological nature can be characterized by $\pi_2(S^2) = \mathbb{Z}$. Since the skyrmion number of the longitudinal mode is 1, we see that $\pi_2(B^+, x_0)$ is equivalent to twice the skyrmion number $\mathfrak{n}_{\mathrm{sk}}$ of the vector characterizing the oriented plane (that is, the longitudinal mode), and it is also equivalent to the Euler number $\mathfrak{e}$ formed by the oriented planes: $\pi_2(B^+, x_0) = 2\mathfrak{n}_{\mathrm{sk}} = \mathfrak{e}$.

Although we have restricted the discussion above to $\pi_2(B^+, x_0)$, the Hamiltonian is in reality characterized by $\pi_2(B, x_0)$. The difference here is that the orientation of the individual $O(1)$ and $O(2)$ sectors are not determined, and only the orientation of the $O(1) \times O(2)$ can be fixed. Because we can always choose to orient the vector bundle, we conclude this section by carrying out a similar discussion for the topological charge $\pi_2(B^-, x_0)$. This can easily be done by performing the transformation $E_{\mathbf{k}} \to E_{\mathbf{k}} R_z(\pi)$, where

$$R_z(\pi) = \begin{pmatrix} -1 & 0 & 0 \\ 0 & -1 & 0 \\ 0 & 0 & 1 \end{pmatrix} \tag{14}$$

is the rotation about the z axis by π. Then, the previous discussion on $\pi_2(B^+, x_0)$ applies without change except that F^+ is now replaced by F^-. Since $R_z(\pi)$ reverses the orientation

of the longitudinal mode and the transverse modes, the transformation reverses the signs of $\mathfrak{e}$ and $\mathfrak{n}_{\rm sk}$. Therefore, we see that by fixing an orientation for the vector bundles, we have $\pi_2(B, x_0) = \pm 2\mathfrak{n}_{\rm sk} = \pm\mathfrak{e}$ (The sign is $+$ if we confine to B^+, while it is $-$ if we confine to B^-). Therefore, the topological charge can be characterized by $\mathfrak{q} = (\mathfrak{n}_{\rm sk}, \mathfrak{e})$, where $\mathfrak{n}_{\rm sk}$ is computed for the longitudinal mode, and $\mathfrak{e}$ is computed for the transverse modes.

To conclude, because we can choose orientation for the fiber bundle in this case, the topological charge can be characterized by the Euler number, which is equivalent to twice the skyrmion number. Also, although the sign of $\mathfrak{n}_{\rm sk}$ and $\mathfrak{e}$ are not determinate in the sense that we can choose the fiber to lie in B^+ or B^-, there is no ambiguity in $\pi_2(B, x_0)$.

Supplementary Note 2. EULER NUMBER AS CHERN NUMBERS OF HELICITY SECTORS

As explained in the main text, we can explain the Euler number for the isotropic phonon Hamiltonian by computing the Chern numbers in the helicity sectors. One way to do this is to introduce a Zeeman coupling along the z direction, $V = L^z h$, where the L^ρ are the usual angular momentum matrices:

$$L^x = \begin{pmatrix} 0 & 0 & 0 \\ 0 & 0 & -i \\ 0 & i & 0 \end{pmatrix}, \tag{15}$$

$$L^y = \begin{pmatrix} 0 & 0 & i \\ 0 & 0 & 0 \\ -i & 0 & 0 \end{pmatrix}, \tag{16}$$

$$L^z = \begin{pmatrix} 0 & -i & 0 \\ i & 0 & 0 \\ 0 & 0 & 0 \end{pmatrix}. \tag{17}$$

Using the degenerate perturbation theory for the transverse modes, the zeroth order eigenstates are $\epsilon_{\mathbf{k},L}$, $\frac{1}{\sqrt{2}}(\epsilon_{\mathbf{k},T_1} + i\epsilon_{\mathbf{k},T_2})$, $\frac{1}{\sqrt{2}}(\epsilon_{\mathbf{k},T_1} - i\epsilon_{\mathbf{k},T_2})$. These states are also eigenstates of the helicity operator $\hat{\mathbf{k}} \cdot \mathbf{L}$ with eigenvalues given respectively by 0, 1, and -1. Note that the Zeeman coupling lifts the degeneracy of the transverse modes, since the lowest corrections to the energy of the transverse modes with helicity 1 and -1 are given by $E_T^{(0)} + h$ and $E_T^{(0)} - h$, where $E_T^{(0)}$ is the energy of the transverse modes without the perturbation. It is straightforward to show that the Berry curvature for each of the helicity sectors with helicity eigenvalues 0, 1, and -1 are 0, $-\frac{\hat{\mathbf{k}}}{k^2}$, and $\frac{\hat{\mathbf{k}}}{k^2}$, respectively. Thus, the Chern numbers for each of the sectors are 0, -2, and 2, respectively. Therefore, the Wilson loop spectrum for the ± 1 helicity sectors should show a winding structure just like the state with $\mathfrak{e} = 2$. Since this remains true in the limit $h \to 0$, we see that the Wilson loop spectrum in the main text can be explained using Chern numbers of the helicity sectors.

Supplementary Note 3. ELASTIC CONTINUUM HAMILTONIAN

In this Supplementary Note, we review the theory of elastic continuum [10, 11].

Convention

We first set down the conventions used in this work for the theory of elasticity. First, the stress tensor σ_{ij} and the strain tensor u_{ij} are related by elastic modulus tensor λ_{ijkl} $(i,j,k,l = x,y,z)$:

$$\sigma_{ij} = \lambda_{ijkl} u_{kl}. \tag{18}$$

Here, the strain and the stress tensors are symmetric,

$$u_{ij} = \frac{1}{2}\left(\frac{\partial u_i}{\partial x_j} + \frac{\partial u_j}{\partial x_i}\right), \quad \sigma_{ij} = \sigma_{ji} \tag{19}$$

where u_i is the displacement along the ith direction, and $x_i = (\mathbf{x})_i = (x,y,z)_i$. The elastic modulus tensor satisfy

$$\lambda_{(ij)(kl)} = \lambda_{(kl)(ij)} = \lambda_{(ji)(kl)} = \lambda_{(ij)(lk)}. \tag{20}$$

Here, we denote the subindices with the parentheses to emphasize the symmetry properties of the elastic modulus tensor. It follows that the elastic modulus tensor λ_{ijkl} has a maximum of 21 independent components. The elastic energy density is given by

$$U[\mathbf{u}] = \frac{1}{2}\lambda_{ijkl} u_{ij} u_{kl}. \tag{21}$$

The Lagrangian density $L[\dot{\mathbf{u}}, \mathbf{u}]$ of the deformed system is expressed as

$$L[\dot{\mathbf{u}}, \mathbf{u}] = \frac{1}{2}\rho \dot{u}_i^2 - U[\mathbf{u}], \tag{22}$$

where $\dot{\mathbf{u}} = \partial_t \mathbf{u}$. Thus, the dynamics of elastic system is described by

$$\rho \ddot{u}_i = \frac{\partial}{\partial x_j}\sigma_{ij}, \tag{23}$$

which can be obtained from the Lagrangian density by using the variational principle. Its Fourier transformation is

$$\omega_{\mathbf{k}}^2 u_i(\mathbf{k}) = \left[\rho^{-1}\lambda_{iljm} k_l k_m\right] u_j(\mathbf{k}) \equiv D(\mathbf{k})_{ij} u_j(\mathbf{k}), \tag{24}$$

where $D(\mathbf{k})$ is called the dynamical matrix. For notational simplicity, we absorb the mass density ρ into the elastic modulus tensor, so that $D(\mathbf{k})_{ij} = \lambda_{iljm} k_l k_m$. Let us note that in the main text, we used the notations $\mathcal{E}_{\mathbf{k}} = \omega_{\mathbf{k}}^2$ and $H_{\mathbf{k}} = D(\mathbf{k})$.

We can simplify Eq. (18) with the help of the Voigt notation:

$$\sigma_1 = \sigma_{xx}, \quad \sigma_4 = \sigma_{yz}, \quad \epsilon_1 = u_{xx} \quad \epsilon_4 = 2u_{yz}, \tag{25}$$
$$\sigma_2 = \sigma_{yy}, \quad \sigma_5 = \sigma_{zx}, \quad \epsilon_2 = u_{yy} \quad \epsilon_5 = 2u_{zx}, \tag{26}$$
$$\sigma_3 = \sigma_{zz}, \quad \sigma_6 = \sigma_{xy}, \quad \epsilon_3 = u_{zz} \quad \epsilon_6 = 2u_{xy}. \tag{27}$$

Now, Eq. (18) becomes $\sigma_I = C_{IJ}\epsilon_J \quad (I,J = 1,\ldots,6)$. In terms of λ_{ijkl}, the elastic tensor C_{IJ} is expressed as

$$C_{IJ} = \begin{pmatrix} \lambda_{xxxx} & \lambda_{xxyy} & \lambda_{xxzz} & \lambda_{xxyz} & \lambda_{xxxz} & \lambda_{xxxy} \\ & \lambda_{yyyy} & \lambda_{yyzz} & \lambda_{yyyz} & \lambda_{xzyy} & \lambda_{xyyy} \\ & & \lambda_{zzzz} & \lambda_{yzzz} & \lambda_{xzzz} & \lambda_{xyzz} \\ & & & \lambda_{yzyz} & \lambda_{xzyz} & \lambda_{xyyz} \\ & & & & \lambda_{xzxz} & \lambda_{xyxz} \\ & & & & & \lambda_{xyxy} \end{pmatrix}_{IJ}. \tag{28}$$

Note that C_{IJ} is symmetric, and we explicitly wrote down only its upper triangular part.

Symmetry properties

For the classification of the elastic continuum Hamiltonian in a 3D crystal, it suffices to consider the 32 crystallographic point groups. To find the constraints due to one of the point groups G, let the matrix representation of an element $\mathcal{G} \in G$ be $\tilde{G}$. Then, λ_{ijkl} satisfies

$$\lambda_{ijkl} = \tilde{G}_{im}\tilde{G}_{jn}\tilde{G}_{ko}\tilde{G}_{lp}\lambda_{mnop}. \tag{29}$$

By imposing the point group symmetries, it is known that there are 9 classes elastic tensors, see Ref. 10. Here, we will focus on the trigonal and the cubic crystal systems.

Trigonal

The point groups $3 = C_3$, $3m = C_{3v}$, $\bar{3} = S_6 = C_{3i}$, $32 = D_3$, and $\bar{3}m = D_{3d}$ belong to trigonal crystal system. There are two classes of elastic tensor C_{IJ} belonging to the trigonal crystal system, depending on the presence of either a twofold rotation symmetry or a mirror symmetry.

(i) Trigonal I: The point groups 3 and $\bar{3}$ lack a twofold rotation symmetry or a mirror symmetry. Then, λ_{ijkl} has 7 independent

elements, which can be organized using the Voigt notation as follows:

$$C_{IJ} = \begin{pmatrix} C_{11} & C_{12} & C_{13} & C_{14} & C_{15} & \\ & C_{11} & C_{13} & -C_{14} & -C_{15} & \\ & & C_{33} & & & \\ & & & C_{44} & & -C_{15} \\ & & & & C_{44} & C_{14} \\ & & & & & \frac{1}{2}(C_{11}-C_{12}) \end{pmatrix}_{IJ} . \tag{30}$$

(ii) Trigonal II: The point groups 32, $3m$, and $\overline{3}m$ have either a twofold rotation symmetry or a mirror symmetry, which kills C_{15}. Then, λ_{ijkl} has 6 independent elements, which can be organized using the Voigt notation as follows:

$$C_{IJ} = \begin{pmatrix} C_{11} & C_{12} & C_{13} & C_{14} & & \\ & C_{11} & C_{13} & -C_{14} & & \\ & & C_{33} & & & \\ & & & C_{44} & & \\ & & & & C_{44} & C_{14} \\ & & & & & \frac{1}{2}(C_{11}-C_{12}) \end{pmatrix}_{IJ} . \tag{31}$$

Cubic

The point groups $23 = T$, $m3 = T_h$, $\overline{4}3m = T_d$, $432 = O$, and $m3m = O_h$ belong to cubic crystal system. λ_{ijkl} has 3 independent elements:

$$C_{IJ} = \begin{pmatrix} C_{11} & C_{12} & C_{12} & & & \\ & C_{11} & C_{12} & & & \\ & & C_{11} & & & \\ & & & C_{44} & & \\ & & & & C_{44} & \\ & & & & & C_{44} \end{pmatrix}_{IJ} . \tag{32}$$

For cubic crystal system, we explicitly write down the dynamical matrix $D(\mathbf{k})$,

$$D(\mathbf{k})_{ij} = \begin{pmatrix} C_{11}k_x^2 + C_{44}(k_y^2+k_z^2) & (C_{12}+C_{44})k_xk_y & (C_{12}+C_{44})k_zk_x \\ & C_{11}k_y^2 + C_{44}(k_z^2+k_x^2) & (C_{12}+C_{44})k_yk_z \\ & & C_{11}k_z^2 + C_{44}(k_x^2+k_y^2) \end{pmatrix} . \tag{33}$$

Supplementary Note 4. K.P HAMILTONIAN

In this Supplementary Note, we consider the k.p Hamiltonian in the presence of the O_h and the T_h point groups. This is done by expanding the Hamiltonian about the triple point using the Gell-Mann matrices,

$$H_{\mathbf{k}} = \sum_n \lambda_n f_n(\mathbf{k}). \tag{34}$$

Because of the $\mathcal{PT}$ symmetry, only λ_n with $n = 1, 3, 4, 6, 8$ are relevant (real symmetric). As before, we denote an element in a point group G by $\mathcal{G}$, and we denote its matrix representation by $\tilde{G}$. The constraint due to $\mathcal{G}$ is $\tilde{G}H_{\mathbf{k}}\tilde{G}^{-1} = H_{\mathcal{G}\mathbf{k}}$. For notational convenience, we define $\Lambda = (\lambda_1, \lambda_3, \lambda_4, \lambda_6, \lambda_8)$.

O_h group: T_{1u}, T_{1g} representations

We find that the T_{1g} representation gives the same constraints, so we explicitly work out only the T_{1u} representation. The transformation properties of Λ are as follows:

$$\begin{aligned}
\tilde{M}_x\Lambda\tilde{M}_x^{-1} &= (-\lambda_1, \lambda_3, -\lambda_4, \lambda_6, \lambda_8),\\
\tilde{M}_y\Lambda\tilde{M}_y^{-1} &= (-\lambda_1, \lambda_3, \lambda_4, -\lambda_6, \lambda_8),\\
\tilde{M}_z\Lambda\tilde{M}_z^{-1} &= (\lambda_1, \lambda_3, -\lambda_4, -\lambda_6, \lambda_8),\\
\tilde{C}_{4z}\Lambda\tilde{C}_{4z}^{-1} &= (-\lambda_1, -\lambda_3, \lambda_6, -\lambda_4, \lambda_8),\\
\tilde{C}_{4x}\Lambda\tilde{C}_{4x}^{-1} &= (\lambda_4, \tfrac{\lambda_3+\sqrt{3}\lambda_8}{2}, -\lambda_1, -\lambda_6, \tfrac{3\lambda_3-\sqrt{3}\lambda_8}{2\sqrt{3}}).
\end{aligned}$$

The symmetry constrained f_n are:

$$\begin{aligned}
f_1 = ak_xk_y, \quad f_3 = b(k_x^2 - k_y^2), \quad f_4 = ak_xk_z,\\
f_6 = ak_yk_z, \quad f_8 = \tfrac{b}{\sqrt{3}}(k_x^2 + k_y^2) - \tfrac{2b}{\sqrt{3}}k_z^2.
\end{aligned} \tag{35}$$

Let us note that this k.p Hamiltonian has the same form as the cubic elastic continuum Hamiltonian in Eq. (33) once we subtract away the trace, with $a = C_{12} + C_{44}$ and $b = \frac{C_{11}}{2} - \frac{C_{44}}{2}$. It is also useful to note that the isotropic elastic continuum Hamiltonian is obtained for $a = 2b$.

O_h group: T_{2u}, T_{2g} representations

We first examine the T_{2u} representation. Here, we take the following matrix representation of the relevant group elements:

$$\tilde{M}_x = \begin{pmatrix} -1 & 0 & 0 \\ 0 & 1 & 0 \\ 0 & 0 & 1 \end{pmatrix}, \quad \tilde{M}_y = \begin{pmatrix} 1 & 0 & 0 \\ 0 & -1 & 0 \\ 0 & 0 & 1 \end{pmatrix},$$
$$\tilde{M}_z = \begin{pmatrix} 1 & 0 & 0 \\ 0 & 1 & 0 \\ 0 & 0 & -1 \end{pmatrix}, \quad \tilde{C}_{4z} = \begin{pmatrix} 0 & -1 & 0 \\ 1 & 0 & 0 \\ 0 & 0 & -1 \end{pmatrix},$$
$$\tilde{C}_{4x} = \begin{pmatrix} -1 & 0 & 0 \\ 0 & 0 & 1 \\ 0 & -1 & 0 \end{pmatrix}. \tag{36}$$

The transformation properties of Λ are as follows:

$$\begin{aligned}
\tilde{M}_x\Lambda\tilde{M}_x^{-1} &= (-\lambda_1, \lambda_3, -\lambda_4, \lambda_6, \lambda_8),\\
\tilde{M}_y\Lambda\tilde{M}_y^{-1} &= (-\lambda_1, \lambda_3, \lambda_4, -\lambda_6, \lambda_8),\\
\tilde{M}_z\Lambda\tilde{M}_z^{-1} &= (\lambda_1, \lambda_3, -\lambda_4, -\lambda_6, \lambda_8),\\
\tilde{C}_{4z}\Lambda\tilde{C}_{4z}^{-1} &= (-\lambda_1, -\lambda_3, -\lambda_6, \lambda_4, \lambda_8),\\
\tilde{C}_{4x}\Lambda\tilde{C}_{4x}^{-1} &= (\lambda_4, \tfrac{\lambda_3+\sqrt{3}\lambda_8}{2}, -\lambda_1, -\lambda_6, \tfrac{3\lambda_3-\sqrt{3}\lambda_8}{2\sqrt{3}}).
\end{aligned}$$

The symmetry constrained f_n are:

$$\begin{aligned}
f_1 = ak_xk_y, \quad f_3 = b(k_x^2 - k_y^2), \quad f_4 = ak_xk_z,\\
f_6 = -ak_yk_z, \quad f_8 = \tfrac{b}{\sqrt{3}}(k_x^2 + k_y^2) - \tfrac{2b}{\sqrt{3}}k_z^2
\end{aligned} \tag{37}$$

Let us note that this Hamiltonian differs from that of T_{1u} only by the transformations $a \to -a$ and $k_x \to -k_x$.

We next examine the T_{2g} representation. Here, we take the following matrix representation of the relevant group elements:

$$\tilde{M}_x = \begin{pmatrix} -1 & 0 & 0 \\ 0 & 1 & 0 \\ 0 & 0 & -1 \end{pmatrix}, \quad \tilde{M}_y = \begin{pmatrix} 1 & 0 & 0 \\ 0 & -1 & 0 \\ 0 & 0 & -1 \end{pmatrix},$$
$$\tilde{M}_z = \begin{pmatrix} -1 & 0 & 0 \\ 0 & -1 & 0 \\ 0 & 0 & 1 \end{pmatrix}, \quad \tilde{C}_{4z} = \begin{pmatrix} 0 & -1 & 0 \\ 1 & 0 & 0 \\ 0 & 0 & -1 \end{pmatrix},$$
$$\tilde{C}_{4x} = \begin{pmatrix} 0 & 0 & 1 \\ 0 & -1 & 0 \\ -1 & 0 & 0 \end{pmatrix}. \tag{38}$$

The action on the Gell-Mann matrices is

$$\begin{aligned}
\tilde{M}_x\Lambda\tilde{M}_x^{-1} &= (-\lambda_1, \lambda_3, \lambda_4, -\lambda_6, \lambda_8),\\
\tilde{M}_y\Lambda\tilde{M}_y^{-1} &= (-\lambda_1, \lambda_3, -\lambda_4, \lambda_6, \lambda_8),\\
\tilde{M}_z\Lambda\tilde{M}_z^{-1} &= (\lambda_1, \lambda_3, -\lambda_4, -\lambda_6, \lambda_8),\\
\tilde{C}_{4z}\Lambda\tilde{C}_{4z}^{-1} &= (-\lambda_1, -\lambda_3, -\lambda_6, \lambda_4, \lambda_8),\\
\tilde{C}_{4x}\Lambda\tilde{C}_{4x}^{-1} &= (\lambda_6, \tfrac{\lambda_3-\sqrt{3}\lambda_8}{2}, -\lambda_4, -\lambda_1, -\tfrac{\sqrt{3}\lambda_3+\lambda_8}{2}).
\end{aligned}$$

The symmetry constrained f_n are:

$$f_1 = ak_xk_y, \quad f_3 = b(k_x^2 - k_y^2), \quad f_4 = ak_yk_z,$$
$$f_6 = ak_xk_z, \quad f_8 = -\tfrac{b}{\sqrt{3}}(k_x^2 + k_y^2) + \tfrac{2b}{\sqrt{3}}k_z^2. \tag{39}$$

Let us note that this Hamiltonian differs from that of the T_{1u} representation only by the transformations $k_x \leftrightarrow k_y$ and $b \to -b$.

T_h group: T_u, T_g representations

Next, let us examine the T_h group. Here, we note that although T, T_h, and T_d groups all support representations with three-fold degeneracy, only the T_h group has inversion symmetry. Because the T_u and the T_g representations of the T_h group yield the same k.p Hamiltonian, we explicitly work out only the T_u representation. We first consider the constraints from T subgroup. The matrix representations of the relevant symmetry elements are

$$\tilde{C}_{2x} = \begin{pmatrix} 1 & 0 & 0 \\ 0 & -1 & 0 \\ 0 & 0 & -1 \end{pmatrix}, \quad \tilde{C}_{2y} = \begin{pmatrix} -1 & 0 & 0 \\ 0 & 1 & 0 \\ 0 & 0 & -1 \end{pmatrix},$$
$$\tilde{C}_{2z} = \begin{pmatrix} -1 & 0 & 0 \\ 0 & -1 & 0 \\ 0 & 0 & 1 \end{pmatrix}, \quad \tilde{C}_3 = \begin{pmatrix} 0 & 0 & 1 \\ 1 & 0 & 0 \\ 0 & 1 & 0 \end{pmatrix}. \tag{40}$$

The transformation properties of Λ are as follows:

$$\tilde{C}_{2x}\Lambda\tilde{C}_{2x}^{-1} = (-\lambda_1, \lambda_3, -\lambda_4, \lambda_6, \lambda_8),$$
$$\tilde{C}_{2y}\Lambda\tilde{C}_{2y}^{-1} = (-\lambda_1, \lambda_3, \lambda_4, -\lambda_6, \lambda_8),$$
$$\tilde{C}_{2z}\Lambda\tilde{C}_{2z}^{-1} = (\lambda_1, \lambda_3, -\lambda_4, -\lambda_6, \lambda_8),$$
$$\tilde{C}_3\Lambda\tilde{C}_3^{-1} = (\lambda_6, \tfrac{-\lambda_3+\sqrt{3}\lambda_8}{2}, \lambda_1, \lambda_4, -\tfrac{\sqrt{3}\lambda_3+\lambda_8}{2}).$$

The constraint is

$$f_1 = ak_xk_y, \quad f_3 = bk_x^2 + ck_y^2 + dk_z^2,$$
$$f_4 = ak_xk_z, \quad f_6 = ak_yk_z,$$
$$f_8 = -\tfrac{2}{\sqrt{3}}[(\tfrac{b}{2} + c)k_x^2 + (\tfrac{c}{2} + d)k_y^2 + (\tfrac{d}{2} + b)k_z^2]. \tag{41}$$

The T_h group additionally has the $\mathcal{S}_6$ symmetry. Its action is $(k_x, k_y, k_z) \to (-k_y, -k_z, -k_x)$ in the momentum space, so that its T_u representation is

$$\tilde{S}_6 = \begin{pmatrix} 0 & -1 & 0 \\ 0 & 0 & -1 \\ -1 & 0 & 0 \end{pmatrix}.$$

The transformation property of Λ under $\mathcal{S}_6$

$$\tilde{S}_6\Lambda\tilde{S}_6^{-1} = (\lambda_4, -\tfrac{\lambda_3+\sqrt{3}\lambda_8}{2}, \lambda_6, \lambda_1, \tfrac{\sqrt{3}\lambda_3-\lambda_8}{2}).$$

Its constraint on f_n is

$$b + c + d = 0. \tag{42}$$

Finally, we remark that although all of the cubic point group symmetries constrain the elastic continuum Hamiltonian in the same way, this is not true for the k.p Hamiltonian. Importantly, we see that the constraint due to O_h and T_h groups are not the same, whereas they give the same constraint on the elastic continuum Hamiltonian.

Supplementary Note 5. LINKING STRUCTURE PROTECTED BY $\mathfrak{q}$

In the main text, we claimed that when the triple point is perturbed such that the triple degeneracy is lifted while keeping the Hamiltonian components to be real, the resulting nodal structure is constrained by $\mathfrak{q}$. To demonstrate this, we plotted the nodal structure in Fig. 3b with the Hamiltonian of the form in Eq. (33) using the parameters $C_{11} = 1.0$, $C_{12} = 0.6$, and $C_{44} = 0.4$, so that $\mathfrak{q} = (1, 2)$. As a comparison with the case in which $\mathfrak{q}$ is not defined, we plotted the nodal structure in Fig. 3c using the same form of the Hamiltonian with the parameters $C_{11} = 1.0$, $C_{12} = 0.2$, and $C_{44} = 2.0$. Both of these Hamiltonians were given a constant perturbation

$$\delta H = 0.02 \times \begin{pmatrix} 2 & -2 & 5 \\ -2 & -4 & 3 \\ 5 & 3 & -2 \end{pmatrix} \tag{43}$$

to plot the nodal structures in Fig. 3d,e. We demonstrate also that the nodal structure has trivial linking structure when $\mathfrak{q} = (0, 0)$ in the main text. For this, acoustic phonons in tellurium (Te) crystal is studied and we add the perturbation δH to the dynamical matrix. As shown in Fig. 5e, the nodal lines are not linked to one another. The dynamical matrix is obtained by using the elastic tensor from Materials Project [12]. Note that the elastic tensor C_{IJ} of Te has the form of Eq. (31). Note that Figs. 3d,e and 5e display the region defined by $-0.28 < k_{x,y,z} < 0.28$.

The purpose of this Supplementary Note is to explain why TATP with $\mathfrak{q} = (1, 2)$ evolves into a nodal ring formed between the highest two bands (L and T_2 modes) threaded by two nodal lines formed between the lowest two bands (T_2 and T_1 modes), as was seen in Fig. 3d. For simplicity, we first assume that the TATP in the isotropic limit is perturbed by a term such as

$$\delta H = \begin{pmatrix} 0 & 0 & 0 \\ 0 & 0 & 0 \\ 0 & 0 & \delta \end{pmatrix}, \tag{44}$$

which preserves the cylindrical symmetry about the z axis.

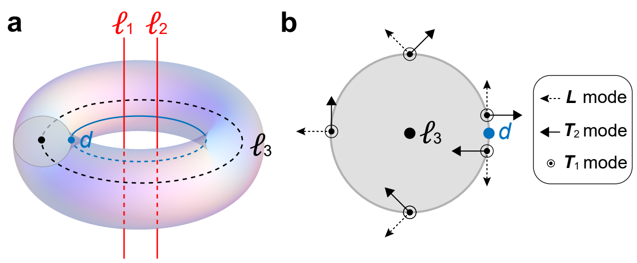

Supplementary Figure 2. **Linking structure.** **a,** ℓ_1 and ℓ_2 are the nodal lines formed between the T_1 and the T_2 modes. ℓ_3 is the nodal ring formed between the L and the T_2 modes is shown in black line. d is the ring of discontinuities in the L mode on the torus. **b,** A cross section of the torus in **a**.

As summarized in Supplementary Figure 2, let ℓ_1 and ℓ_2 (red lines) denote the two nodal lines formed between the T_1 and T_2 modes, and let ℓ_3 (black ring) denote the nodal ring formed between the L and the T_2 modes. Note that we are assuming that $\mathcal{E}_{\mathbf{k},L} \geq \mathcal{E}_{\mathbf{k},T_2} \geq \mathcal{E}_{\mathbf{k},T_1}$. Although the lines ℓ_1 and ℓ_2 overlap in the momentum space due to the cylindrical symmetry, we draw them separately for clarity.

The goal is to show that ℓ_1 and ℓ_2 should penetrate ℓ_3. First, we note that ℓ_3 is protected by the π-Berry phase. Because of the π-Berry phase, the L mode ($\epsilon_{\mathbf{k},L}$) around the nodal ring ℓ_3 shows winding structure as shown in Supplementary Figure 2b. Importantly, there is a discontinuity in $\epsilon_{\mathbf{k},L}$ due to the π Berry phase [6], indicated by a blue dot and labeled as d in Supplementary Figure 2b. Notice that this is compatible with the skyrmion texture of $\epsilon_{\mathbf{k},L}$ on a sphere surrounding ℓ_3. In fact, because of the skyrmion texture, $\epsilon_{\mathbf{k},L}$ on all 2D slices of the torus have similar wavefunction texture. In particular, the wavefunction discontinuity indicated with a blue dot in Supplementary Figure 2b forms a circle, as shown in Supplementary Figure 2a as a blue line and labeled as d.

Now, because ℓ_3 becomes a 2D Dirac point on the 2D slice (grey cut in Supplementary Figure 2), the T_2 mode ($\epsilon_{\mathbf{k},T_2}$) has the texture schematically shown in Supplementary Figure 2b. Therefore, the T_1 mode ($\epsilon_{\mathbf{k},T_1}$), being orthogonal to both L_1 and T_2 modes, is tangential to the nodal ring ℓ_3. It follows that just above the blue line, we have

$$\begin{aligned} \epsilon_{\mathbf{k},L} &= (0,0,1) \\ \epsilon_{\mathbf{k},T_2} &= \tfrac{1}{k}(-k_x,-k_y,0) \\ \epsilon_{\mathbf{k},T_1} &= \tfrac{1}{k}(-k_y,k_x,0). \end{aligned} \tag{45}$$

Because the first two components of T_1 and T_2 modes have vorticity of 2, by which we mean that they are eigenstates of the Hamiltonian of the form $H_{\mathbf{k}} \propto 2k_x k_y \sigma_x + (k_x^2 - k_y^2)\sigma_z$, there must be two Dirac points at $k_z = 0$, which corresponds to the two nodal lines ℓ_1 and ℓ_2 threading the nodal ring ℓ_3.

This geometric proof can also be generalized to the case in which the cylindrical symmetry is broken. We only give a sketch of the proof since it does not give us further intuition. We first consider a surface D whose boundary is ℓ_3. Then, consider an arbitrarily small circular path c surrounding ℓ_3. On this path, we choose gauge such that $\epsilon_{\mathbf{k},L}$ has discontinuity at the point d at which the circular path c intersects the surface D. Note that just above and below d, $\epsilon_{\mathbf{k},L}$ point in the opposite directions due to the π Berry phase provided by ℓ_3. Also, as we trace c, $\epsilon_{\mathbf{k},L}$ and $\epsilon_{\mathbf{k},T_2}$ must lie on the same plane: otherwise, $\epsilon_{\mathbf{k},T_1}$, which is orthogonal to $\epsilon_{\mathbf{k},L}$ and $\epsilon_{\mathbf{k},T_2}$, will not converge to a single vector as we shrink the radius of c to zero. Now, let us consider $\epsilon_{\mathbf{k},L}$ just above d. As we trace ℓ_3, $\epsilon_{\mathbf{k},L}$ traces a closed loop on a unit sphere. Now, consider a fixed orthonormal frame (a fixed set of orthonormal vectors) $\mathcal{F}_{\text{fix}}$: ($\hat{\mathbf{x}}_{\text{fix}}$, $\hat{\mathbf{y}}_{\text{fix}}$, $\hat{\mathbf{z}}_{\text{fix}}$). We can define a local frame (orthonormal vectors as a function of $\mathbf{k}$ along the loop ℓ_3) $\mathcal{F}_{\mathbf{k},\text{loc}}$: ($\hat{\mathbf{x}}_{\mathbf{k},\text{loc}}$, $\hat{\mathbf{y}}_{\mathbf{k},\text{loc}}$, $\hat{\mathbf{z}}_{\mathbf{k},\text{loc}}$) along ℓ_3 by transforming $\mathcal{F}_{\text{fix}}$ such that $\hat{\mathbf{z}}_{\mathbf{k},\text{loc}}$ aligns with $\epsilon_{\mathbf{k},L}$ (e.g. rotate the fixed frame about the axis normal to $\epsilon_{\mathbf{k},L}$ and $\hat{\mathbf{z}}_{\text{fix}}$ to obtain $\mathcal{F}_{\mathbf{k},\text{loc}}$). Then, the $\epsilon_{\mathbf{k},T_1}$ and the $\epsilon_{\mathbf{k},T_2}$ modes expressed in the local frame must have vorticity

2. (Note that expressing $\epsilon_{\mathbf{k},T_1}$ and $\epsilon_{\mathbf{k},T_2}$ in this local frame is basically the same as deforming the $\epsilon_{\mathbf{k},L}$ modes along ℓ_3 just above d to align with $\hat{\mathbf{z}}_{\rm fix}$ in the fixed frame. Since $\epsilon_{\mathbf{k},L}$ mode has skyrmion texture, $\epsilon_{\mathbf{k},T_1}$ and $\epsilon_{\mathbf{k},T_2}$ must have vorticity 2.)

We have thus shown that the skyrmion texture of the L mode forces there to be Dirac points with total vorticity 2 in the ring ℓ_3. There are many other ways to show why this should be true. For example, we can similarly prove the linking structure by assuming that $\mathfrak{e} = 2$ for the T_1 and T_2 modes, and showing that the L mode on the D-shaped closed path (e.g. begin from south pole in Supplementary Figure 1, go straight to the north pole, and back to the south pole along the surface of the sphere) has π-Berry phase. Alternatively, one can use the quaternion charge method as in Ref. 13, or use the Dirac-string formulation in Ref. 7.

Supplementary Note 6. NODAL LINES

Convention for counting nodal lines

Because of the Euler number $\mathfrak{e}$ of the transverse modes defined on a sphere S^2 enclosing the triple point, there must be 2D Dirac points on S^2 with total vorticity of $-2\mathfrak{e}$. Because the low-energy Hamiltonian $\propto k^2$, these Dirac points form a nodal line in the 3D momentum space. By the number of nodal lines *emanating* from the triple point at $\mathbf{k} = 0$, we mean the number of Dirac points on the the sphere S^2. Because two nodal lines emanating from the triple point can be naturally paired, when we refer to the number of nodal lines without any qualifications, we mean the number of paired nodal lines emanating from the triple point. Note that when the dispersion of the Dirac points on the sphere is quadratic, the number of Dirac points is 2.

Because of the relation $\mathfrak{e} = -\frac{N_t}{2}$ [7], $\mathfrak{e}$ places a constraint on the total vorticity N_t of the Dirac points on S^2. Here, the vorticity is defined by writing the effective Hamiltonian around the Dirac point as $H_D = r(\mathbf{k})\cos\theta(\mathbf{k})\sigma_x + r(\mathbf{k})\sin\theta(\mathbf{k})\sigma_z$, and counting the winding number of $(\cos\theta(\mathbf{k}), \sin\theta(\mathbf{k}))$. Therefore, when $\mathfrak{e} = 2$, there must be at least 4 Dirac points on the sphere, i.e. at least 4 nodal lines must emanate from the triple point.

Nodal lines in k.p Hamiltonian of O_h group and the elastic continuum Hamiltonian in cubic crystals

Here, we study the nodal structure of the k.p Hamiltonian supporting three-dimensional representation for the O_h group, or equivalently, the nodal structure in the elastic continuum Hamiltonian for the cubic crystal system. Recall that the traceless Hamiltonian takes the form in Eq. (34) with f_n given as in Eq. (35). Although it is not practical to analytically diagonalize the Hamiltonian at a generic $\mathbf{k}$, we can gain an understanding of the nodal structure by diagonalize it along the high symmetry lines. Now, we assume that $a \neq 0$, so that there is only one parameter $\frac{b}{a}$ that determines the form of the Hamiltonian. Along the line with $k_y = k_z = 0$, the eigenvalues are $\frac{4bk_x^2}{3a}$, $\frac{-2bk_x^2}{3a}$, $\frac{-2bk_x^2}{3}$, and along the line with $k_x = k_y = k_z$, the eigenvalues are $2k_x^2$, $-k_x^2$, $-k_x^2$. From this, we can conclude that for $\frac{b}{a} < 0$, $\mathfrak{q}$ cannot be defined because along the line with $k_x = k_y = k_z$, the lower two bands are degenerate, while for the line with $k_y = k_z = 0$, the upper two bands are degenerate. On the other hand, for $\frac{b}{a} > 0$, the behavior along the high symmetry lines suggest that $\mathfrak{q}$ can be defined, which we have confirmed through a numerical study in a reasonable parameter range. Since we obtain the isotropic case for $b = \frac{a}{2}$, we conclude that $\mathfrak{q} = (1, 2)$ for $\frac{b}{a} \in \mathbb{R}^+$. These results are summarized in Supplementary Figure 3a.

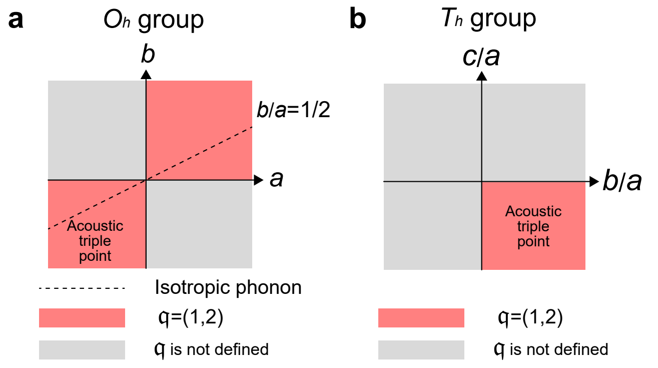

Supplementary Figure 3. **Topological phase diagram. a, b,** Phase diagram for point groups O_h (**a**) and T_h (**b**).

Now, let us relate the condition $b/a > 0$ to the Born stability condition of phonons. Recall that in terms of the elastic constants, $a = C_{12} + C_{44}$ and $b = \frac{C_{11}}{2} - \frac{C_{44}}{2}$. Also recall that the Born stability conditions are $C_{44} > 0$, $C_{11} - C_{12} > 0$, $C_{11} + 2C_{12} > 0$. First, let us note that it is not possible for both $a < 0$ and $b < 0$, which would imply that $a + 2b = C_{11} + C_{12} < 0$. This contradicts the Born stability conditions, which constrain $(C_{11} - C_{12}) + 2(C_{11} + 2C_{12}) = 3(C_{11} + C_{12}) > 0$. Therefore, there are two possibilities (excluding fine-tuned situations where $a = 0$ or $b = 0$): (1) $a > 0$ and $b > 0$ and (2) $ab < 0$. It is clear from the above analysis that for the case (1), the longitudinal velocity exceeds the transverse velocity along the two families of high symmetry lines. For the case (2), it is clear from the above analysis that when $a > 0$ and $b < 0$, the longitudinal velocity exceeds the transverse velocity along the line $k_x = k_y = k_z$, but not along the line $k_y = k_z = 0$. For the case (2), it is also possible that $a < 0$ and $b > 0$, in which case we just have a reverse situation where the transverse velocity exceeds the longitudinal velocity along the line $k_x = k_y = k_z$, but not along the line $k_y = k_z = 0$.

Nodal lines in k.p Hamiltonian of T_h group

For the k.p Hamiltonian with the T_u and T_g representations of the T_h group, the Hamiltonian is given by Eq. (34) with f_n given by Eqs. (41) and (42). As in the case with the O_h group, we assume that $a \neq 0$, so that the Hamiltonian is determined by two parameters, $\frac{b}{a}$ and $\frac{c}{a}$ [Note that we can eliminate d using Eq. (42)]. Since it is not practical to analytically diagonalize the Hamiltonian at a generic $\mathbf{k}$, we first study the energy spectrum along the high symmetry lines. Along $k_x = k_y = 0$, the eigenvalues are $-\frac{2}{3a}(2b+c)k_z^2$, $\frac{2}{3a}(b + 2c)k_z^2$, and $\frac{2}{3a}(b - c)k_z^2$. Along $k_x = k_y = k_z$, the eigenvalues are $2k_x^2$, $-k_x^2$, $-k_x^2$. Thus, we expect the phase boundaries to be located at $\frac{b+c}{a} = 0$, $\frac{b}{a} = 0$, and $\frac{c}{a} = 0$ (these are the parameters for which two of the energy bands along $k_x = k_y = k_z$ become equal). Through a numerical study, we find that $\mathfrak{q} = (1, 2)$ when $\frac{b}{a} > 0$ and $\frac{c}{a} < 0$, as illustrated in Supplementary Figure 3b. Here, we note that this is consistent with the result for the O_h group, since the the Hamiltonian reduces to that of the O_h group (with T_{1u} representation) when $c = -b$.

Supplementary Note 7. DETAILS OF THE 3D LIEB LATTICE MODEL

In this Supplementary Note, we give the details of the 3D Lieb lattice model discussed in the main text.

Tight-binding Hamiltonian

The lattice structure is shown in Fig. 4a in the main text. In each unit cell, there are 4 sublattice sites located at $\mathbf{x}_1 = (0,0,0)$, $\mathbf{x}_2 = (1/2,0,0)$, $\mathbf{x}_3 = (0,1/2,0)$, $\mathbf{x}_4 = (0,0,1/2)$. The tight-binding Hamiltonian is given by

$$H_{\rm Lieb}(\mathbf{k}) = H_0(\mathbf{k}) + H_1(\mathbf{k}) + H_2(\mathbf{k}) + H_3(\mathbf{k}),$$

$$H_0(\mathbf{k}) = \begin{pmatrix} \epsilon_1 & 0 & 0 & 0 \\ 0 & \epsilon_2 & 0 & 0 \\ 0 & 0 & \epsilon_2 & 0 \\ 0 & 0 & 0 & \epsilon_2 \end{pmatrix}, \quad H_1(\mathbf{k}) = 2t_0 \begin{pmatrix} 0 & \cos\frac{k_x}{2} & \cos\frac{k_y}{2} & \cos\frac{k_z}{2} \\ \cos\frac{k_x}{2} & 0 & 0 & 0 \\ \cos\frac{k_y}{2} & 0 & 0 & 0 \\ \cos\frac{k_z}{2} & 0 & 0 & 0 \end{pmatrix},$$

$$H_2(\mathbf{k}) = 4t_1 \begin{pmatrix} 0 & 0 & 0 & 0 \\ 0 & 0 & \cos\frac{k_x}{2}\cos\frac{k_y}{2} & \cos\frac{k_z}{2}\cos\frac{k_x}{2} \\ 0 & \cos\frac{k_x}{2}\cos\frac{k_y}{2} & 0 & \cos\frac{k_y}{2}\cos\frac{k_z}{2} \\ 0 & \cos\frac{k_z}{2}\cos\frac{k_x}{2} & \cos\frac{k_y}{2}\cos\frac{k_z}{2} & 0 \end{pmatrix},$$

$$H_3(\mathbf{k}) = 2\left(\cos k_x + \cos k_y + \cos k_z\right) \begin{pmatrix} t_2 & 0 & 0 & 0 \\ 0 & t_3 & 0 & 0 \\ 0 & 0 & t_3 & 0 \\ 0 & 0 & 0 & t_3 \end{pmatrix}. \tag{46}$$

Here, ϵ_1 is the onsite potential for the s orbital located at $\mathbf{x}_1$, and ϵ_2 is the onsite potential for the s orbitals located at $\mathbf{x}_2$, $\mathbf{x}_3$, and $\mathbf{x}_4$. t_0, t_1, and t_2 are the hopping amplitudes for the nearest, the second-nearest, and the third-nearest neighbors. We note that for the band structure in the main text, we have chosen the parameters $\epsilon_1 = -2.0$, $\epsilon_2 = 1.2$, $t_0 = 1.0$, $t_1 = 0.3$, $t_2 = 0.2$, and $t_3 = 0.1$.

Effective Hamiltonian of the triple point

The tight-binding Hamiltonian $H_{\rm Lieb}(\mathbf{k})$ has the O_h point group symmetry and the time-reversal symmetry $\mathcal{T}$. As was noted in the main text, the band structure exhibits a triple point at $R = (\pi,\pi,\pi)$. This triple point is protected by the C_{2x}, C_{2y}, and $C_{3[111]}$ symmetries [14].

By making an analogy between the triple point at R and the acoustic phonon, we can map the highest energy band to the longitudinal phonon mode and the lower two energy bands to the transverse phonon modes by explicitly computing the effective Hamiltonian $H_{\rm eff}(\mathbf{k})$ near the triple point using the Löwdin partitioning [15]:

$$H_{\rm eff}(\mathbf{k})_{nm} = h(\mathbf{k}_0)_{nm} + \sum_{i=1}^{3} h_i(\mathbf{k}_0)_{nm} k_i + \sum_{i,j=1}^{3} \left[\frac{1}{2} h_{ij}(\mathbf{k}_0)_{nm} + \sum_{\overline{m}\neq n,m} \frac{h_i(\mathbf{k}_0)_{n\overline{m}} h_j(\mathbf{k}_0)_{\overline{m}m}}{E_n(\mathbf{k}_0) - E_{\overline{m}}(\mathbf{k}_0)} \right] k_i k_j + O(k^3), \tag{47}$$

$$h(\mathbf{k}_0)_{ab} = \langle a,\mathbf{k}_0 | H_{\rm Lieb}(\mathbf{k}_0) | b,\mathbf{k}_0 \rangle, \quad h_i(\mathbf{k}_0)_{ab} = \langle a,\mathbf{k}_0 | (\partial_i H_{\rm Lieb})(\mathbf{k}_0) | b,\mathbf{k}_0 \rangle, \quad h_{ij}(\mathbf{k}_0)_{ab} = \langle a,\mathbf{k}_0 | (\partial_i \partial_j H_{\rm Lieb})(\mathbf{k}_0) | b,\mathbf{k}_0 \rangle, \tag{48}$$

where $\mathbf{k}_0 = (\pi,\pi,\pi)$, and the indices n, m run over the bands that form the triple point, while $\overline{m}$ runs over the other bands, the lowest band in this case. Also, the band indices a, b run over any band.

Straightforward computation yields

$$H_{\text{eff}}(\mathbf{k}) = \begin{pmatrix} t_3(k^2-6)+\frac{t_0^2}{6t_2-\epsilon_1}k_x^2+\epsilon_2 & (t_1+\frac{t_0^2}{6t_2-\epsilon_1})k_xk_y & (t_1+\frac{t_0^2}{6t_2-\epsilon_1})k_zk_x \\ (t_1+\frac{t_0^2}{6t_2-\epsilon_1})k_xk_y & t_3(k^2-6)+\frac{t_0^2}{6t_2-\epsilon_1}k_y^2+\epsilon_2 & (t_1+\frac{t_0^2}{6t_2-\epsilon_1})k_yk_z \\ (t_1+\frac{t_0^2}{6t_2-\epsilon_1})k_zk_x & (t_1+\frac{t_0^2}{6t_2-\epsilon_1})k_yk_z & t_3(k^2-6)+\frac{t_0^2}{6t_2-\epsilon_1}k_z^2+\epsilon_2 \end{pmatrix} \tag{49}$$

$$= \big(t_3(k^2-6)+\epsilon_2\big)\,\mathbb{1}_3 - t_1\text{Diag}(k_x^2,k_y^2,k_z^2) + \left(t_1+\frac{t_0^2}{6t_2-\epsilon_1}\right)(k_x,k_y,k_z)^T(k_x,k_y,k_z). \tag{50}$$

We can therefore decompose $H_{\text{eff}}(\mathbf{k})$ as

$$H_{\text{eff}}(\mathbf{k}) = (\epsilon_2-6t_3)\mathbb{1}_3 + D_{\text{eff}}(\mathbf{k}), \tag{51}$$

$$D_{\text{eff}}(\mathbf{k}) = \begin{pmatrix} C_{11}k_x^2+C_{44}(k_y^2+k_z^2) & (C_{12}+C_{44})k_xk_y & (C_{12}+C_{44})k_zk_x \\ (C_{12}+C_{44})k_xk_y & C_{11}k_y^2+C_{44}(k_z^2+k_x^2) & (C_{12}+C_{44})k_yk_z \\ (C_{12}+C_{44})k_zk_x & (C_{12}+C_{44})k_yk_z & C_{11}k_z^2+C_{44}(k_x^2+k_y^2) \end{pmatrix}, \tag{52}$$

where

$$C_{11} = \frac{t_0^2}{6t_2-\epsilon_1}+t_3, \quad C_{12} = t_1+\frac{t_0^2}{6t_2-\epsilon_1}-t_3, \quad C_{44} = t_3. \tag{53}$$

It is crucial to note that $D_{\text{eff}}(\mathbf{k})$ is identical to the dynamical matrix in cubic symmetric system. In this way, we can directly compare the triple point in the electronic system and that in the elastic material.

Supplementary Note 8. THE EFFECT OF BOUNDARY CONDITION ON SURFACE LOCALIZED STATES

In this Supplementary Note, we first study the implications of Dirichlet boundary condition, which is sometimes employed when studying the surface states of topological insulators. As a comparison, we also review the free boundary condition which is known to yield surface acoustic waves.

Dirichlet boundary condition

Since there is no direct analog of the 'free boundary condition' of phonons in electronic systems, we first study the Dirichlet boundary condition. In the bulk, the equation of motion is

$$i\frac{\partial}{\partial t}\psi = (-a\nabla^2 - b\nabla^T\nabla)\psi. \tag{54}$$

We introduce a boundary at $z = 0$ and study waves propagating along the k_x direction ($k_y = 0$). To solve this problem, let us first impose some symmetry constraints. The mirror symmetry

$$M_y = \begin{pmatrix} 1 & 0 & 0 \\ 0 & -1 & 0 \\ 0 & 0 & 1 \end{pmatrix} \tag{55}$$

gives $\psi_y = 0$. Further, we impose

$$C_{2z}T = \begin{pmatrix} 1 & 0 & 0 \\ 0 & 1 & 0 \\ 0 & 0 & -1 \end{pmatrix}\mathcal{K}, \tag{56}$$

so that ψ_x is purely real and ψ_z is purely imaginary. Then, the wavefunction naturally satisfy the Hermiticity condition, which is often imposed when solving for the Fermi arc of Weyl semimetal:

$$\begin{aligned} 0 = \big[& a\left(\langle\nabla_z\psi_2, \psi_1\rangle - \langle\psi_2, \nabla_z\psi_1\rangle\right) \\ & + b\left(\nabla_z\psi_{2z}^*\psi_{1z} - \psi_{2z}^*\nabla_z\psi_{1z}\right) \\ & - ibk_x\left(\psi_{2z}^*\psi_{1x} + \psi_{2x}^*\psi_{1z}\right)\big]_{z=0}. \end{aligned} \tag{57}$$

It is convenient to separate the wavefunction into the transverse and the longitudinal modes. Note that this is possible since we can expand any wavefunction by linear combination of the transverse and the longitudinal modes. For the transverse part we write

$$\psi_T = \begin{pmatrix} \psi_{Tx} \\ \psi_{Ty} \\ \psi_{Tz} \end{pmatrix} e^{\kappa_T z} e^{ik_x x - i\omega t}, \tag{58}$$

where κ_T is the inverse decay length, and $\psi_{T(x,y,z)}$ are constants. The transversality condition $\nabla \cdot \psi_T = 0$ gives

$$ik_x\psi_{Tx} + \kappa_T\psi_{Tz} = 0. \tag{59}$$

Also, the equation of motion in the bulk gives

$$\kappa_T = \sqrt{k_x^2 - \omega/a}. \tag{60}$$

Thus,

$$\psi_T = c_T\begin{pmatrix} \kappa_T \\ 0 \\ -ik_x \end{pmatrix} e^{ik_x x - i\omega t} e^{\kappa_T z}. \tag{61}$$

Similarly, the longitudinal part is written as

$$\psi_L = \begin{pmatrix} \psi_{Lx} \\ \psi_{Ly} \\ \psi_{Lz} \end{pmatrix} e^{\kappa_L z} e^{ik_x x - i\omega t}. \tag{62}$$

The longitudinal condition $\nabla \times \psi_L = 0$ gives

$$\kappa\psi_{Lx} - ik_x\psi_{Lz} = 0. \tag{63}$$

Also, the equation of motion in the bulk gives

$$\kappa_L = \sqrt{k_x^2 - \omega/(a+b)}. \tag{64}$$

Thus,

$$\psi_L = c_L\begin{pmatrix} k_x \\ 0 \\ -i\kappa_L \end{pmatrix} e^{ik_x x - i\omega t} e^{\kappa_L z}. \tag{65}$$

The Dirichlet condition is that $\psi = 0$ at the boundary. This requires the following two equations to be satisfied:

$$c_T\sqrt{k_x^2 - \omega/a} + c_L k_x = 0 \tag{66}$$

$$c_T k_x + c_L\sqrt{k_x^2 - \omega/(a+b)} = 0. \tag{67}$$

Thus, we must have

$$-\frac{k_x^2}{\sqrt{k_x^2 - \omega/a}} + \sqrt{k_x^2 - \omega/(a+b)} = 0. \tag{68}$$

This yields two solutions

$$\begin{aligned} \omega &= 0 \\ \omega &= k_x^2(2a+b). \end{aligned} \tag{69}$$

When the first condition is satisfied, Eq. (67) demands $k_x = 0$, so that the solution is not really independent of $\omega = k_x^2(2a + b)$.

Let us note that the following conditions must be satisfied to obtain surface-localized states (i.e. $\kappa_{T,L} > 0$):

$$-\frac{b}{a} > 1 \quad \text{and} \quad \frac{b}{a+b} > 1. \tag{70}$$

The parameter region satisfying these conditions is shown in Supplementary Figure 4a.

Although the Dirichlet boundary condition is sometimes used to show that surface localized states exist in topologi-

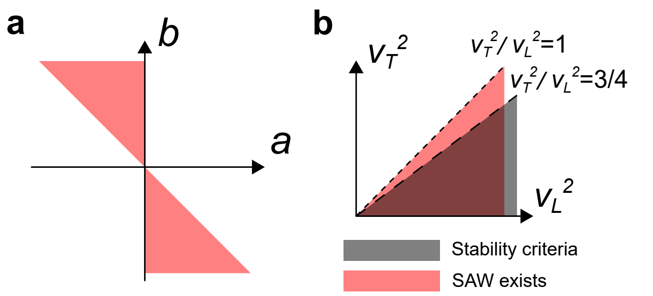

Supplementary Figure 4. **Conditions for the presence of surface localized states. a,** Diagram showing when surface localized states can appear for Dirichlet boundary condition. **b,** Diagram showing when surface localized states can appear for free boundary condition. Note that when the phonon satisfies the stability criteria, surface acoustic wave always appears.

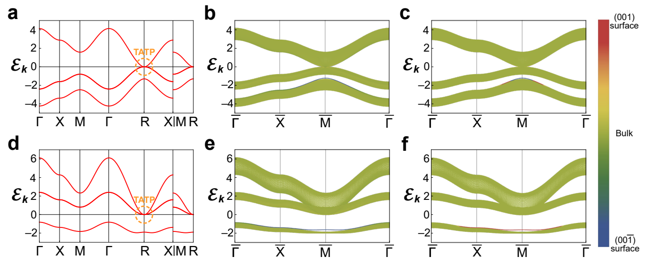

Supplementary Figure 5. **Absence of surface states in 3D Lieb model. a,** The band structure of the 3D Lieb model in the isotropic limit with the parameters $\epsilon_1 = 0.5$, $\epsilon_2 = -1.2$, $t_0 = 1.0$, $t_1 = 0.0$, $t_2 = 0.3$, and $t_3 = -0.2$. Note that two bands corresponding to transverse phonon modes are degenerate. The parameters are chosen such that if we impose the Dirichlet boundary condition, surface localized modes appear. **b,** Surface spectrum for 001 termination. Color indicates the center of wavefunction. Note that the finite-size slab is constructed by stacking 20 layers along the z direction. **c,** Same as **b** but we remove the dangling atoms so that the finite-size slab is mirror symmetric in the z-direction. **d,** The band structure with the $\epsilon_1 = 0.5$, $t_0 = 1.0$, $t_1 = 0.0$, $t_2 = 0.4$, $t_3 = 0.2$. The parameters are chosen that $a = 0.2$, $b = 0.5263$ in Eq. (72) to mimic the isotropic phonon. Notice that there are no surface localized modes analogous to the surface acoustic waves in **e** and **f**, which are the surface spectrums for asymmetric and symmetric termination, respectively, in the 001 direction.

cal insulators, we note that naively solving for the wavefunctions in a finite size system does not yield surface modes consistent with the Dirichlet boundary condition. To illustrate this, let us study the Lieb lattice with the parameters chosen such that the triple point at R falls in the region in which the Dirichlet boundary condition yields surface localized modes, see Eq. (70).

We first notice from Eq. (50) that the isotropic limit can be achieved by setting $t_1 = 0$ and $\epsilon_2 = 6t_3$. In order to test the existence of surface states, we choose the parameter values, $\epsilon_1 = 0.5$, $\epsilon_2 = -1.2$, $t_0 = 1.0$, $t_1 = 0.0$, $t_2 = 0.3$, $t_3 = -0.2$. The band structure along the high symmetry lines is shown in Supplementary Figure 5a. We note that the second and third lowest bands are degenerate throughout the BZ. The k.p Hamiltonian near R is given by

$$H(\mathbf{k}) = -0.2k^2 \mathbb{1}_3 + 0.77(k_x, k_y, k_z)^T(k_x, k_y, k_z), \quad (71)$$

which corresponds to Eq. (54) with $a = -0.2$ and $b = 0.77$. From Eq. (70), we expect surface states for Dirichlet boundary condition. However, we do not observe the desired surface states in the surface spectrum with both asymmetric and symmetric terminations (Supplementary Figure 5b,c). In fact, it is not clear how Dirichlet boundary condition can be achieved for discrete tight-binding model.

We note in passing that for phonon, the equation of motion is

$$-\frac{\partial^2 \psi}{\partial t^2} = (-a\nabla^2 - b\nabla^T\nabla)\psi. \quad (72)$$

Thus, the only difference is that $\omega \to \omega^2$, and $a = v_T^2$, $b = v_L^2 - v_T^2$. We see that there are no localized states for the Dirichlet boundary condition for phonons (Note that we always have $v_L > v_T$ for isotropic phonon).

Free boundary condition

For completeness, we show that surface acoustic waves appear in isotropic medium by following Ref. 11. It is well known that isotropic phonons support surface acoustic waves. Denoting the stress tensor by σ_{ij}, the free boundary condition is $\sum_k \sigma_{ij} n_j = 0$, where $\mathbf{n} = (0, 0, 1)$, since we are assuming a boundary at $z = 0$. This entails the following: $\sigma_{xz} = \sigma_{yz} = \sigma_{zz} = 0$. For isotropic medium, we have $\sigma_{ij} = K u_{kk}\delta_{ij} + 2\mu(u_{ij} - \frac{1}{3}\delta_{ij}u_{kk})$, where $u_{ij} = \frac{1}{2}(\frac{\partial u_i}{\partial x_j} + \frac{\partial u_j}{\partial x_i})$ is the strain tensor. Here, the K and μ are related to the longitudinal and transverse velocity by the relations $K = \lambda + \frac{2}{3}\mu$, $\mu = v_T^2$, and $\lambda = v_L^2 - 2v_T^2$. Thus, $\sigma_{yz} = 0$ implies $u_{yz} = 0$. Since we assume the that the wave does not depend on the value of y, this implies $\frac{\partial u_y}{\partial z} = 0$. Using the surface wave ansatz, we obtain $u_y = 0$.

Next, we note that the transverse and longitudinal modes essentially take the form in Eqs. (61) and (65), respectively. With this in mind, we now examine the other two constraints. The condition $\sigma_{xz} = 0$ implies $u_{xz} = 0$, which translates to $c_T(k_x^2 + \kappa_T^2) + 2c_L k_x \kappa_L = 0$. The condition $\sigma_{zz} = 0$ gives $2c_T v_T^2 \kappa_T k_x + c_L(2v_T^2 k_x^2 + v_L^2(\kappa_L^2 - k_x^2)) = 0$. These two combines to $4k_x^2 \kappa_L \kappa_T = (k_x^2 + \kappa_T^2)^2$. Thus, ω and k_x satisfy $\frac{\omega^8}{v_T^8} - 8k_x^2\frac{\omega^6}{v_T^6} + 8k_x^4\frac{\omega^4}{v_T^4}(3 - 2\frac{v_T^2}{v_L^2}) + 16k_x^6\frac{\omega^2}{v_T^2}(\frac{v_T^2}{v_L^2} - 1) = 0$. We then use the ansatz $\omega = v_T k_x \xi$ to obtain $\xi^6 - 8\xi^4 + 8\xi^2(3 - 2\frac{v_T^2}{v_L^2}) + 16(\frac{v_T^2}{v_L^2} - 1) = 0$.

Let us note that the localization requires $1 - \xi^2 > 0$ and $1 - \frac{v_T^2}{v_L^2}\xi^2 > 0$. Also, the SAW at (k'_x, k'_y) is $R(\phi)u_{\text{SAW}}(\mathbf{k}')$, where $\mathbf{k}' = R(\phi)\mathbf{k}$, where R is rotation by ϕ about the z axis. For isotropic case, the longitudinal velocity always exceeds the transverse velocity due to the Born stability criterion that $v_T^2/v_L^2 < \frac{3}{4}$. Since the SAW exists for $v_T^2/v_L^2 < 1$, we see

that isotropic medium always has SAW for free boundary, as summarized in Supplementary Figure 4b. However, this may not be true when we move away from isotropic case, and it would be interesting to investigate the properties of surface acoustic waves in relation to the topology.

Finally, let us note because the equation of motion for phonon is $\frac{\partial^2 \mathbf{u_k}}{\partial t^2} = -H(\mathbf{k})\mathbf{u_k}$ and the equation of motion electron is $i\frac{\partial \psi_{\mathbf{k}}}{\partial t} = H(\mathbf{k})\psi_{\mathbf{k}}$, the wavefunction are eigenvectors of $H(\mathbf{k})$. However, the free boundary condition for phonon does not have a clear interpretation for electrons, and we are not guaranteed to obtain surface modes. We demonstrate this using the Lieb lattice example. As before, we choose the parameters such that the k.p Hamiltonian near R can be mapped to isotropic phonon. Here, we additionally demand $v_L > v_T > 0$ under this identification. The parameters $\epsilon_1 = 0.5$, $t_0 = 1.0$, $t_1 = 0.0$, $t_2 = 0.4$, $t_3 = 0.2$ yield $a = 0.2$, $b = 0.5263$ in Eq. (72). The energy bands along the high symmetry lines are shown in Supplementary Figure 5d. Although the isotropic phonon should show surface localized states, we do not find any for the electronic case, as can be seen in Supplementary Figure 5e,f for both asymmetric and symmetric terminations.

Supplementary Note 9. PHONON ANGULAR MOMENTUM AT THE SURFACE

Because both the phonon angular momentum Hall effect and the surface acoustic wave are properties of the low-energy phonons, it is tempting to ask whether there is any relation between them. It is known that surface acoustic waves induce rotational motion [16], and we find that surface acoustic wave also has phonon angular momentum. The reason behind this is that the inversion symmetry is broken whenever surface is introduced, as we explain below.

Let $\mathbf{u}(\mathbf{r})$ be the displacement and $\mathbf{p}(\mathbf{r})$ be the momentum of elastic medium at position $\mathbf{r}$. For convenience, we choose to work with the rescaled position and momentum, $\mathbf{u}(\mathbf{r})\sqrt{\rho} \to \mathbf{u}(\mathbf{r})$ and $\mathbf{p}/\sqrt{\rho} \to \mathbf{p}(\mathbf{r})$, where ρ is the mass density. We will always put $\hbar = 1$. In the momentum space, the low-energy phonon Hamiltonian (elastic continuum approximation) is $\mathcal{H}^{(0)} = \frac{1}{2}\sum_{\mathbf{k}} \mathbf{x}_{\mathbf{k}}^\dagger H_{\mathbf{k}} \mathbf{x}_{\mathbf{k}}$, where $\mathbf{x}_{\mathbf{k}} = \left(\begin{smallmatrix}\mathbf{p}_{\mathbf{k}}\\ \mathbf{u}_{\mathbf{k}}\end{smallmatrix}\right)$ and

$$H_{\mathbf{k}} = \begin{pmatrix} 1_{3\times 3} & 0 \\ 0 & D_{\mathbf{k}} \end{pmatrix}. \tag{73}$$

Here, $(D_{\mathbf{k}})_{\alpha\beta}$ is the dynamical matrix.

The phonon angular momentum refers to the orbital motion of the atoms making up the solid with respect to the rest position. For elastic continuum, we have $L = \int d\mathbf{r}\, \mathbf{u}(\mathbf{r}) \times \mathbf{p}(\mathbf{r})$. In the momentum space, $\mathcal{L}^\rho = \frac{1}{2}\sum_{\mathbf{k}} \mathbf{x}_{\mathbf{k}}^\dagger L^\rho \mathbf{x}_{\mathbf{k}}$, where

$$L^\rho = \begin{pmatrix} 0 & -\ell^\rho \\ \ell^\rho & 0 \end{pmatrix}, \quad \ell^\rho_{\mu\nu} = \epsilon_{\mu\nu\rho}, \tag{74}$$

and $\epsilon_{\mu\nu\rho}$ is the Levi-Civita symbol (we drop the zero-point phonon angular momentum as it is not our focus). Since the time-reversal symmetry and inversion symmetry are represented by

$$\mathcal{T} = \begin{pmatrix} -1_{3\times 3} & 0 \\ 0 & 1_{3\times 3} \end{pmatrix} \mathcal{K}, \quad \mathcal{P} = -1_{6\times 6}, \tag{75}$$

we see that the phonon angular momentum transforms like an axial vector in the momentum space. Therefore, when both the time reversal and inversion symmetry are present, the phonon angular momentum vanish in the momentum space.

Let us now consider the surface acoustic wave in an isotropic medium propagating in the x direction with surface termination at $z = 0$. Then, the mirror symmetry $\mathcal{M}_y : (x, y, z) \to (x, -y, z)$ forces the angular momentum to point along the y direction. To calculate this component of the angular momentum, we normalize the polarization vectors such that $\int_{-\infty}^{0} dz\, |\epsilon(k_x, z)|^2 = 1$. Then the angular momentum density of the surface acoustic wave at z in the y direction is given by $L_{\text{SAW}}(k_x, z) = -i\epsilon(k_x, z)^\dagger \ell^y \epsilon(k_x, z)$. For concreteness, let us assume that $v_T/v_L = 0.5$, so that the surface acoustic wave has energy spectrum $E = kv_T\xi$ with $\xi \approx 0.933$, see Free boundary condition in Supplementary Note 8. We show $L_{\text{SAW}}(k_x, z)$ in Supplementary Figure 6a.

Using the isotropicity of the phonon under consideration, the surface acoustic wave with $\mathbf{k} = (k_x, k_y)$ therefore carries angular momentum $\mathbf{L}_{\text{SAW}}(\mathbf{k}, z) = L_{\text{SAW}}(k, z)\hat{\mathbf{z}} \times \hat{\mathbf{k}}$ as shown in Supplementary Figure 6c. The winding structure of the phonon angular momentum is reminiscent of the spin texture in Rashba electron gas, and we can expect that there is a thermal version of the Rashba-Edelstein effect [17, 18] for surface acoustic waves. Note, however, that the bulk modes (non-localized) must also satisfy the boundary condition, so that it is not possible to neglect their contribution.

To see how much of the surface angular momentum results from SAW under the application of a thermal gradient, we compute the angular momentum induced by thermal gradient in a two-dimensional square lattice, as shown in Supplementary Figure 6d (here, it suffices to consider only the in-plane vibration). Here, we use the mass-spring model with the nearest neighbor longitudinal and transverse spring constants, and the next nearest neighbor longitudinal and transverse spring constants. Let $D_{\mathbf{k}}$ be the resulting $2N \times 2N$ dynamical matrix, where N is the thickness, so that

$$H_{\mathbf{k}} = \begin{pmatrix} 1_{2N\times 2N} & 0 \\ 0 & D_{\mathbf{k}} \end{pmatrix}. \tag{76}$$

It is also convenient to use the notation $|n, \mathbf{k}\rangle$ for phonon eigenstates, which satisfy

$$\sigma^y H_{\mathbf{k}} |n, \mathbf{k}\rangle = E_{\mathbf{k},n} |n, \mathbf{k}\rangle, \quad \langle n, \mathbf{k}|\sigma^y|m, \mathbf{k}\rangle = (\sigma^z)_{nm}. \tag{77}$$

Here, σ^i with $i = x, y, z$ are the generalized Pauli matrices with block structure of $H_{\mathbf{k}}$ in Eq. (76). Note that there are $2N$ eigenstates with $n = -2N, -1, 1, ..., 2N$.

Let us apply a thermal gradient along the y direction and compute the phonon angular momentum induced in the z direction. For a rough estimation of the thermally induced phonon angular momentum, we use the Boltzmann transport theory with constant phonon lifetime τ. Then, the phonon angular momentum density induced by temperature gradient $(\nabla_y T)\hat{\mathbf{y}}$ is $\langle L_x^z \rangle_{\text{neq}} - \langle L_x^z \rangle_{\text{eq}} = -\lambda_y(x)\nabla_y T$, where

$$\lambda_y(x) = \frac{\tau}{2k_B T^2} \frac{1}{V} \sum_{\mathbf{k}} \sum_{n=-2N}^{2N} \langle n, \mathbf{k}|L_x^z|n, \mathbf{k}\rangle \times \langle n, \mathbf{k}|v_{\mathbf{k},y}|n, \mathbf{k}\rangle \frac{\sigma^z_{n,n} E_{\mathbf{k},n} e^{\sigma^z_{nn} E_{\mathbf{k},n}/k_B T}}{(e^{\sigma^z_{nn} E_{\mathbf{k},n}/k_B T} - 1)^2}. \tag{78}$$

Here, T is the temperature, k_B is the Boltzmann constant. L^z is as given in Eq. (74) with $\rho = z$ for each of the N blocks (corresponding to each of the position x in Supplementary Figure 6), except that μ and ν takes only the values x and y, since we are studying a two-dimensional model.

We show $\lambda_y(x)$ in Supplementary Figure 6d. Because the lowest two energy bands (near $k_y = 0$) are the surface acoustic waves for each of the two edges, we estimate the surface acoustic wave contribution by summing over the two lowest energy states ($n = 1, 2$) and the hole partners ($n = -1, -2$). We see that the angular momentum due to the surface acoustic waves quickly decays, in contrast to that resulting from the bulk states, which does not decay to 0 as fast. We also see

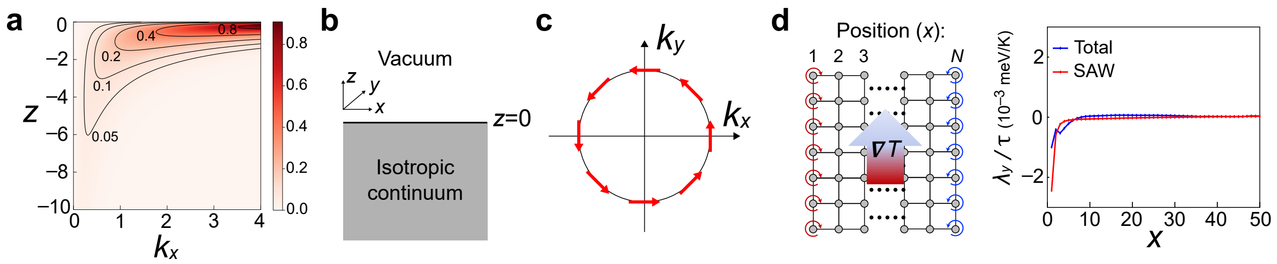

Supplementary Figure 6. **Phonon angular momentum of surface acoustic waves. a,** Distribution of the angular momentum of the surface acoustic wave at given k_x for the infinite slab geometry shown in **b**. The color represents the density of angular momentum along the y direction. Because the sum over z of the angular momentum along the y direction for fixed k_x is positive, we conclude that the angular momentum of the surface acoustic wave at a fixed energy is given as shown in **c**. **d,** Left: square lattice model. The nearest neighbor longitudinal and transverse spring constants are 50 and 20, respectively. The next nearest longitudinal and transverse spring constants are 12.5 and 5.0, respectively. Right: By applying thermal gradient along the y direction, there is an accumulation of phonon angular momentum at the edges. For calculation, the total thickness was set to 300. We show the distribution near the left edge (the distribution of angular momentum is antisymmetric in the x direction.)

that the bulk states also contribute significantly to the surface angular momentum, although the trend of the total angular momentum induced on the surface is similar to that due to the SAW.

SUPPLEMENTARY REFERENCES

[1] Bouhon, A. *et al.* Non-Abelian reciprocal braiding of Weyl points and its manifestation in ZrTe. *Nature Physics* **16**, 1137–1143 (2020).
[2] Bouhon, A., Bzdušek, T. & Slager, R.-J. Geometric approach to fragile topology beyond symmetry indicators. *Physical Review B* **102**, 115135 (2020).
[3] Ünal, F. N., Bouhon, A. & Slager, R.-J. Topological Euler class as a dynamical observable in optical lattices. *Physical Review Letters* **125**, 053601 (2020).
[4] Hatcher, A. *Algebraic Topology* (Cambridge University Press, 2002).
[5] Itō, K. *Encyclopedic dictionary of mathematics*, vol. 1 (MIT press, 1993).
[6] Ahn, J., Kim, D., Kim, Y. & Yang, B.-J. Band topology and linking structure of nodal line semimetals with Z_2 monopole charges. *Physical Review Letters* **121**, 106403 (2018).
[7] Ahn, J., Park, S. & Yang, B.-J. Failure of Nielsen-Ninomiya theorem and fragile topology in two-dimensional systems with space-time inversion symmetry: Application to twisted bilayer graphene at magic angle. *Physical Review X* **9**, 021013 (2019).
[8] Hatcher, A. *Vector Bundles and K-Theory* (unpublished, 2003).
[9] Bott, R. & Tu, L. W. *Differential forms in algebraic topology*, vol. 82 (Springer Science & Business Media, 2013).
[10] Nye, J. F. *et al. Physical properties of crystals: their representation by tensors and matrices* (Oxford university press, 1985).
[11] Lifshitz, E., Kosevich, A. & Pitaevskii, L. *Theory of elasticity* (Butterworth-Heinemann, 1986).
[12] De Jong, M. *et al.* Charting the complete elastic properties of inorganic crystalline compounds. *Scientific data* **2**, 1–13 (2015).
[13] Tiwari, A. & Bzdušek, T. Non-abelian topology of nodal-line rings in $\mathcal{PT}$-symmetric systems. *Physical Review B* **101**, 195130 (2020).
[14] Bradlyn, B. *et al.* Beyond Dirac and Weyl fermions: Unconventional quasiparticles in conventional crystals. *Science* **353** (2016).
[15] Löwdin, P.-O. A note on the quantum-mechanical perturbation theory. *The Journal of Chemical Physics* **19**, 1396–1401 (1951).
[16] Matsuo, M., Ieda, J., Harii, K., Saitoh, E. & Maekawa, S. Mechanical generation of spin current by spin-rotation coupling. *Physical Review B* **87**, 180402 (2013).
[17] Bychkov, Y. A. & Rashba, É. I. Properties of a 2D electron gas with lifted spectral degeneracy. *JETP Lett.* **39**, 78 (1984).
[18] Edelstein, V. M. Spin polarization of conduction electrons induced by electric current in two-dimensional asymmetric electron systems. *Solid State Commun.* **73**, 233–235 (1990).
